# Unveiling the Role of Furanic Compounds in Coffee Quality

**DOI:** 10.1021/acsomega.5c13418

**Published:** 2026-04-08

**Authors:** Valentina Chaves Tognocchi, Aline de Oliveira Garcia, Fernanda F. G. Dias, Silvia Amélia Verdiani Tfouni, Wellington da Silva Oliveira

**Affiliations:** † Institute of Food Technology (ITAL), Avenida Brasil 2880, 13070-178 Campinas, SP, Brazil; ‡ Centro de Ciência e Qualidade de Alimentos, Instituto de Tecnologia de Alimentos—ITAL, Avenida Brasil n 2880, 13070-178 Campinas, SP, Brazil; § Department of Food Science and Nutrition, University of Minnesota, St. Paul, Minnesota 55108, United States

## Abstract

Furan and its derivatives are generated during coffee roasting. These compounds contribute to both potential toxicological risks and sensory characteristics of the final beverage. Despite their relevance, few analytical approaches allow the simultaneous quantification of key furanic compounds, such as furan (FU), furfural (FURF), 5-methylfurfural (5-MF), and furaneol (FNOL) in coffee. Moreover, the relationship between their concentrations and the coffee quality has not been fully elucidated. In this study, a multiple headspace gas chromatography–mass spectrometry (MHS–GC–MS/MS) method was optimized for the quantification of these compounds. Using only 20 mg of coffee, the method achieved detection and quantification limits ranging from 0.004 to 0.2 mg·kg^–1^ and 0.012 to 0.6 mg·kg^–1^, respectively, with precision below 20%. The validated method was applied to 20 commercial coffee samples, which also underwent sensory evaluation and an overall quality classification. Results indicated a positive correlation between coffee acidity and furanic compound levels, with higher concentrations of these compounds being associated with improved sensory quality. Nonetheless, strategies remain necessary to maintain desirable flavor attributes while minimizing consumer exposure to these potentially harmful compounds.

## Graphical abstract

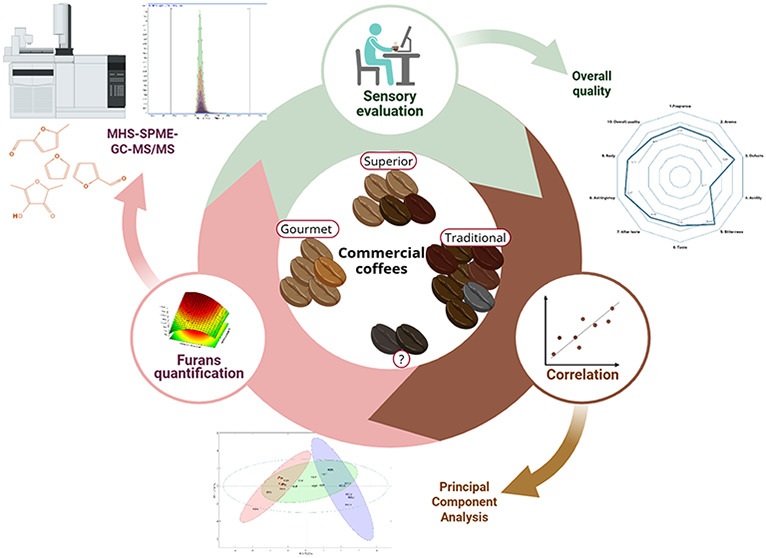

## 1 Introduction

Coffee is one of the most popular beverages worldwide,^1^ valued for its unique flavor and high caffeine content.^2^ Although coffee itself is classified as noncarcinogenic, the roasting process can lead to the formation of several potentially toxic compounds^3^ including furan (FU), furfural (FURF), furaneol (FNOL), and 5-methylfurfural (5-MF). Furan (FU) is a thermal food processing contaminant reported in several food products^4^ and classified by the International Agency for Research on Cancer as possibly carcinogenic to humans (group 2B).^5^ Coffee is considered one of the major dietary sources of furan exposure in adults and the elderly, according to EFSA.^6^ In contrast, FNOL and FURF are key aroma-active compounds responsible for caramel-like notes. However, both were associated with the generation of reactive oxygen species, which may induce DNA damage and cytotoxic effects.^7^ Unlike these compounds, 5-MF has no safety concerns reported regarding its use as a flavoring in food^8^ and has instead been applied as a chemical marker for assessing coffee quality^9^ or distinguishing coffees based on variety and geographical origin.^10^

While various technological strategies have been investigated to predict or reduce the formation of toxic furanic compounds during roasting,^11,12^ these compounds also contribute to the sensory acceptance of coffee, as they are associated with desirable sweet and caramel-like aromas characteristic of roasted coffee. Therefore, although they represent a potential toxicological risk, their presence is considered essential for the quality and pleasant flavor profile of high-grade coffees.^13^ Despite their dual importance, no studies to date have directly linked the concentrations of furanic compounds to the sensory characteristics of commercial coffee samples.

Several techniques for quantification of furanic compounds have been reported in the literature, including liquid chromatography-tandem mass spectrometry (LC-MS/MS),^14^ gas chromatography-tandem mass spectrometry (GC-MS/MS),^15^ ultraperformance liquid chromatography coupled to an ultraviolet detector (UHPLC-UV),^16^ gas chromatography with flame ionization detector,^17^ among others.^4,18^ However, the simultaneous determination of FU, FURF, 5-MF, and FNOL in coffee remains a challenge due to their distinct chemical properties. These compounds differ widely in volatility, polarity, and stability, requiring tailored extraction and analytical conditions. As a result, conventional approaches often fail to provide consistent recovery and accurate quantification across all four furanic compounds.

Solid-phase microextraction (SPME) has become one of the most popular equilibrium-based techniques for sampling and sample preparation in analytical and bioanalytical chemistry.^19^ SPME can integrate sampling, extraction, concentration, and sample introduction into a single solvent-free step, coupled to a gas chromatography system.^20^ However, the technique does not perform an exhaustive extraction of the sample, which may compromise the compounds’ quantification.^21^

To overcome these issues, multiple headspace (MHS) SPME offers an effective alternative. This technique efficiently extracts volatile compounds from samples while minimizing matrix effect, evaporation losses, and sample manipulation.^22^ In MHS, several extractions are performed on the same sample, promoting an exhaustive extraction of the analytes. Thus, based on the exponential decay of each analyte and the total peak area, it is possible to quantify each compound.^21^ Notably, no studies to date have reported the simultaneous quantification of furanic compounds in coffee by using MHS.

Considering the analytical challenges associated with quantifying furanic compounds, the present study aimed to optimize and validate a method for the simultaneous quantification of four furanic compounds in commercial coffee samples using MHS-SPME extraction followed by GC-MS/MS. The validated method was used on a set of 20 commercial samples. Results obtained were used to investigate the correlation between the concentrations of these compounds and the sensory quality of the coffee, providing new insights into the toxicological and sensory relevance of these compounds in commercial coffee.

## 2 Materials and Methods

### 2.1 Samples

Twenty roasted ground coffee samples were collected from stores in Campinas, SP, Brazil. Samples were kept in their original packaging and stored at room temperature until analysis. The samples were labeled as follows: S1–S8 - traditional coffee, S9–S14 - superior coffee, and S15–S20 - gourmet coffee (Table S1).

### 2.2 Reagents and Standards

Standards of FU (TCI America, 99% purity), FUR (Sigma, 99%), 5-MF (Sigma, ≥98.5%), FNOL (Sigma, ≥99%), hexyl acetate (Sigma, >98%), and dibutyl phthalate (Sigma, 99%) were used in this study. Chromatographic grade methanol and hexane were obtained from Merck. The relevant chemical information for each analyte is listed in Table 1.

**Table 1:**
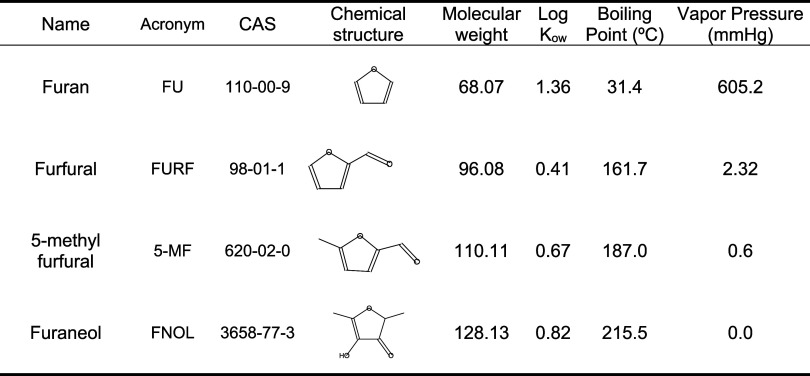
Name, Acronym, CAS Number, Chemical Structure, Molecular Weight, Log K_ow_, Boiling Point, and Vapor Pressure of the Evaluated Analytes

Stock solutions (1000 mg·kg^–1^) were prepared in methanol for FNOL and in hexane for FU, FUR, and 5-MF. From the stock solution, solutions containing 100 mg·kg^–1^ of each analyte were prepared using the same solvents. Subsequently, calibration solutions containing the limit of quantification (LOQ), 2, 6, 10, 14, 18, and 22 mg·kg^–1^ were prepared in water for FNOL and in dibutyl phthalate for FU, FUR, and 5-MF. Hexyl acetate (1 mg·kg^–1^) diluted in dibutyl phthalate was used as an internal standard.

### 2.3 Instrumental Conditions

A gas chromatography system (Agilent 8890 GC coupled with an Agilent 7010B triple quadrupole mass spectrometer) equipped with a PAL (RSI 85) automatic sampler and a multimode injector (MMI) was used for the analysis. A high-efficiency electron ionization (EI) source (Agilent) was employed for the ionization. Separation was achieved on a VF-WAXms polar column (60 m × 0.25 mm × 0.50 μm, Agilent). The oven temperature was set at 34 °C for 1 min, then increased at a rate of 2 °C min^–1^ to 40 °C and maintained for 8 min. Subsequently, the temperature was raised at 10 °C min^–1^ to 240 °C and held for 12 min, resulting in a total analysis time of 44 min. The MMI was held at 25 °C for 0.01 min and then ramped at 650 °C min^–1^ to 300 °C and maintained until the end of the run.

To optimize multiple-reaction monitoring (MRM), a solution containing 10 mg·L^–1^ of each compound in methanol was used. For this purpose, Mass Hunter optimizer software was used to determine the precursor ions, product ions, and the collision energy of each compound evaluated. The MRM condition obtained was used for the next steps of optimization.

Finally, the selected SPME optimal conditions were as follows: incubation time of 30 min, extraction time of 30.5 min, agitation of 587.5 rpm, and 80 °C incubation temperature.

### 2.4 Experimental Design

After MRM optimization, an experimental design was performed to optimize the extraction conditions by MHS-SPME. A central composite rotational design (CCRD), consisting of 28 trials, was carried out to optimize the incubation time (minutes, *X*_1_), extraction time (minutes, *X*_2_), extraction temperature (°C, *X*_3_), and agitation (rpm, *X*_4_) (Table 2). A CAR/PDMS/DVB fiber (1 cm) was used in the extraction

**Table 2:** Experimental Design for Optimizing the Simultaneous Extraction of Furanic Compounds Using Headspace Solid-Phase Microextraction (HS-SPME)

Coded Values					
variables	–2	–1	0	1	2
*X*_1_: incubation time (min)	20	30	40	50	60
*X*_2_: extraction time (min)	20	30	40	50	60
*X*_3_: extraction temperature (°C)	65	70	75	80	85
*X*_4_: agitation (rpm)	250	362.5	475	587.5	700

Real Values				
trial	incubation time (minutes)	extraction time (minutes)	incubation temperature (°C)	agitation (rpm)
1	30	30	70	362
2	50	30	70	362
3	30	50	70	362
4	50	50	70	362
5	30	30	80	362
6	50	30	80	362
7	30	50	80	362
8	50	50	80	362
9	30	30	70	587
10	50	30	70	587
11	30	50	70	587
12	50	50	70	587
13	30	30	80	587
14	50	30	80	587
15	30	50	80	587
16	50	50	80	587
17	40	40	75	250
18	40	40	75	700
19	40	40	65	475
20	40	40	85	475
21	40	20	75	475
22	40	60	75	475
23	20	40	75	475
24	60	40	75	475
25	40	40	75	475
26	40	40	75	475
27	40	40	75	475
28	40	40	75	475

Subsequently, the Derringer and Suich^23^ method was applied to select the best extraction conditions that would maximize the extraction of all analytes. A mix of standards prepared in water containing 1 mg·kg^–1^ of each compound was used in both optimization steps.

The impact of sample size (100, 60, and 20 mg) and the number of extractions for the MHS extraction were also evaluated. Sample size is crucial for MHS extraction as a very small amount of sample can undergo significant mass loss between the first and subsequent extractions, preventing exponential decay and consequently affecting sample quantification. On the other hand, large sample amounts can cause headspace saturation, hindering the exponential decay.^21^ Therefore, it is necessary to determine the ideal sample amount to allow for exponential decay and compound quantification. The sample amount selected was used for the validation steps.

### 2.5 Quantification of Furan Compounds

Twenty milligrams of each sample was weighed into a 20 mL vial and subjected to five consecutive extractions. The total area of each compound was obtained using eq 1

1 $$A_{T}=\sum_{i=1}^{i\to \infty }A_{i}=\frac{A_{1}}{1-e^{-q}}=\frac{A_{1}}{1-\beta }$$

where A_T_ is the estimated total peak area, *A*_1_ is the peak area of the first extraction, and q is a constant describing the exponential decay associated with β.

The constant β is given by the slope of the regression curve of the logarithms of the areas of the individual peaks as a function of the number of extractions, according to eq 2

2 $$\ln A_{i}=\ln A_{1}+\left(i-1\right)\cdot \ln\beta$$

where *A_i_* is the area obtained in the *i*th extraction. This linear equation is represented as *y* = *ax* + *b*, where ln *A*_1_ is the *y*-axis intercept, and ln β is the slope.

Since the MHS eliminates the matrix effect,^24^ the concentration of each compound was determined using external calibration curves. For this, calibration solutions were subjected to five consecutive multiple headspace extractions using the same conditions. Hexyl acetate was used as an internal standard on a standard-in-fiber preloading, which was performed by exposing the fiber to the headspace at 80 °C for 2 min. This procedure reduces errors associated with adding a standard to the sample and allows monitoring the reliability and efficiency of the SPME during use.^14^

Analytical curves were prepared in triplicate. The concentrations of each compound in the samples were determined using the total area obtained in the first extraction along with the β term and eq 1.

### 2.6 Method Validation

Validation parameters included limits of detection (LOD) and quantification (LOQ), linearity, and precision (intraday and interday). LOD was determined as 3 times the signal-to-noise ratio. LOQ was determined as the lowest concentration that resulted in the decay of the analytes and a suitable β value. The linearity was evaluated using seven-point calibration curves (LOQ, 2, 6, 10, 14, 18, and 22 mg·kg^–1^) injected in triplicate. The linearity was assessed by ANOVA. Intraday and interday precision were determined from three different points on the calibration curves (LOQ, midpoint, and highest point on the curve). Precision was expressed as the relative standard deviation of replicate measurements at each level. The validated method was used to quantify furanic compounds in 20 commercial coffee samples.

### 2.7 Sensory Analysis

In Brazil, roasted coffee is classified at national and regional levels based on sensory and physicochemical characteristics. At a regional level, the São Paulo State Department of Agriculture and Supply (SAA-SP) adopts a technical descriptive approach to sensory classification. Considering overall quality (OQ), coffees are classified as specialty (8.0 ≤ OQ ≤ 10), gourmet (7.3 ≤ OQ ≤ 7.9), superior (6.0 ≤ OQ ≤ 7.2), traditional (4.5 ≤ OQ ≤ 5.9), and not recommended for consumption (OQ < 4.5).^25^

To verify the classification indicated on the coffee labels and evaluate the relationship between the levels of furans and quality standards, the samples were subjected to a sensory analysis by nine trained experts. The sensory panel was part of the coffee quality program certified by the Brazilian Coffee Industry Association.^26^ All panelists provided informed consent after receiving a detailed explanation of the study, and participation could be discontinued at any time, in accordance with the guidelines of the Research Ethics Committee.

For this assessment, the brewed coffee was prepared by percolation using a #103 paper filter, 50 g of roasted coffee, and 500 mL of mineral water at 92 °C. The samples were stored in insulated bottles during the analysis and were served at 70 °C.

Odor, defects, acidity, bitterness, flavor, aftertaste, astringency, and body of the coffee beverage, along with the fragrance of the coffee powder, were evaluated to determine the overall coffee quality. All attributes were assessed using a 0–10 cm unstructured line scale, following the method described by Domingues, Ferreira, Garcia, and Morgano.^27^ The overall quality score was used to classify each sample as specialty, gourmet, superior, traditional, or not recommended for consumption, according to the guidelines from São Paulo State Department of Agriculture and Supply.^25^ The sensory data were correlated with the concentrations of furanic compounds using principal component analysis (PCA).

## 3 Results and Discussion

During the roasting process of green coffee beans, carbohydrates, organic acids, phenolic compounds, lipids, proteins, free amino acids, and other minor substances are converted into compounds responsible for the odor and flavor of roasted coffee.^12^ Maillard reaction, caramelization, and pyrolysis are the primary reaction pathways responsible for forming these compounds; however, they also lead to the formation of FU, FURF, 5-MF, and FNOL. Dietary exposure to furanic compounds may pose public health concerns, and agencies, such as the FDA^28^ and EFSA,^6,29^ monitor or have established limits for these compounds in specific food categories. However, methods to simultaneously quantify these compounds in coffee remain scarce. Therefore, an optimized MHS-SPME-GC-MS/MS was developed as an alternative for their concurrent quantification and is described below.

### 3.1 Experimental Design

Extraction using SPME relies on kinetic and thermodynamic processes in a multiphasic system involving the equilibria between the sample and the headspace (*K*_sh_), as well as between the headspace and the polymeric coating (*K*_hf_).^19^ *K*_hf_ or *K*_sh_ can be affected by several factors, including incubation time (*X*_1_), extraction time (*X*_2_), extraction temperature (*X*_3_), and agitation time (*X*_4_). Therefore, in SPME-based methods, these parameters must be carefully optimized to improve extraction efficiency and enhance the overall sensitivity of the analytical protocol.

Table 3 shows the compounds evaluated, along with their retention times, precursor ions (*m*/*z*), product ions (*m*/*z*), and collision energies used in this study. The most intense transition was used for quantification, and the second most intense was used for confirmation.

**Table 3:** Multiple-Reaction Monitoring (MRM) Transitions Used for the Quantification of Furanic Compounds

compounds	retention time (min)	precursor ion (*m*/*z*)	product ion (*m*/*z*)	collision energy (V)
furan	6.4	68	39	25
			29	25
hexyl acetate^a^	18.22	84	41	15
			69	0
furfural	25.65	96	39	40
			68	10
5-methylfurfural	27.23	110	53	20
			81	10
furaneol	32.39	128	43	20
			85	5

a Internal standard.

The increase of the extraction temperature (°C) negatively impacted FU, FURF, and 5-MF extraction (Figures S1A and 1B,1C) while no significant impact was observed for FNOL. These compounds have a higher vapor pressure than FNOL (Table 1), therefore, their concentration in headspace may be higher than in the solid sample at the evaluated temperatures. Consequently, an increase in temperature during equilibrium shifts the distribution coefficient between the fiber and the headspace toward the headspace, reducing the efficiency of the extraction process.^30^

**Figure 1:**
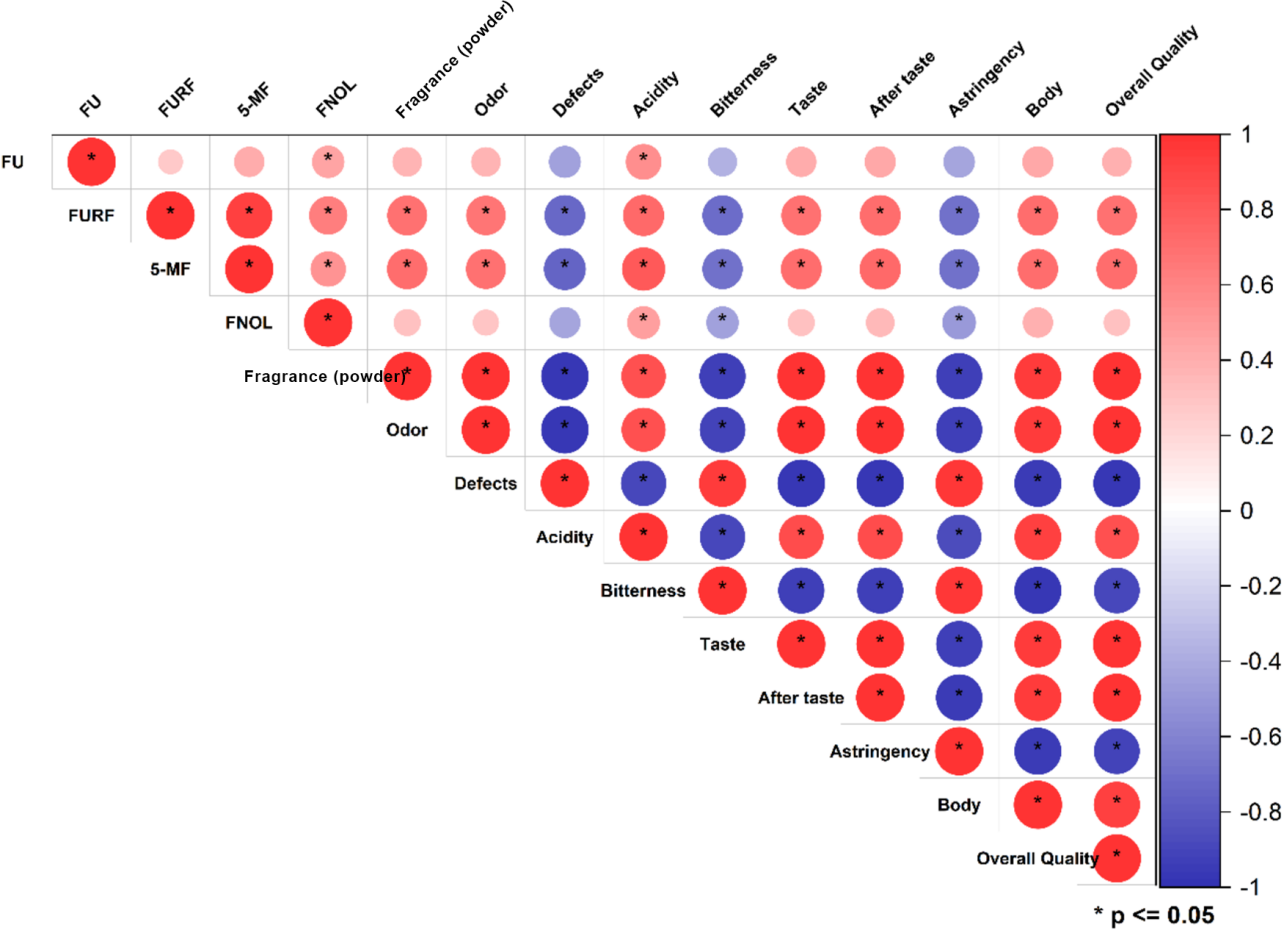
Correlation analysis of furanic compounds concentration and sensory parameters in 20 commercial coffee samples. *Indicates statistically significant correlation at *p* < 0.05.

Higher incubation times and stirring also negatively affected the FU extraction. For FURF and 5-MF (Figure S1B,C), longer extraction times also had a negative impact, resulting in lower responses. On the other hand, longer extraction times showed a positive effect on FU and 5-MF (Figure S1A–C). For FNOL, only stirring showed a positive effect during extraction. FNOL exhibits a higher boiling point than the other compounds evaluated and is more polar, requiring higher temperatures and stirring to shift the equilibrium to headspace and increase extraction by the SPME fiber.

The maximum sensitivity of SPME is achieved under equilibrium conditions, which depend on the specific characteristics of each compound, such as the diffusion coefficient and distribution constant of the analyte, and the thickness of the extraction phase.^31^ In multianalyte extraction methods, it is common for optimal extraction conditions to differ among analytes. For SPME, these variations can arrive from differences in physicochemical properties such as log *P* and Henry’s constant.^32^ These parameters strongly influence how each compound partitions within the food matrix and transitions from the sample into the headspace, thereby affecting the extraction efficiency. In this study, higher extraction temperatures reduced the adsorption of FU, FURF, and 5-MF; however, it was required for the FNOL extraction. Additionally, the agitation and extraction time affected the evaluated compounds differently. To achieve the optimal overall response, the Derringer and Suich desirability function was employed to simultaneously maximize the extraction efficiency of all compounds.^23^ Derringe and Suich are based on a desirability function that evaluates each response on a scale of 0 to 1, where 0 represents a less desirable value and 1 represents a more desirable value. A model was proposed combining individual responses to maximize each compound extraction.^33^ The optimal SPME conditions were 30 min of incubation at 80 °C, 30.5 min of extraction, and 587.5 rpm of agitation.

### 3.2 MHS-SPME Optimization

The initial step to optimize MHS-SPME methods is to determine the appropriate sample mass necessary to perform multiple extractions. To identify the optimal mass, aliquots of 100, 60, and 20 mg were evaluated (Figure S2). The 60 and 100 mg samples showed a good coefficient of determination (*R* ^2^) and β values varying from 0.71 to 0.97. The 20 mg sample showed β values between 0.71 and 0.94 and higher angular coefficients (Table S2), indicating a greater sensitivity to concentration changes.

For proper MHS, the β value must be ideally between 0.4 and 0.95. Very large samples often fail to show significant decay during extractions, making it impossible to calculate the β value.^34^ Conversely, values below 0.4 indicate that the MHS is not needed, since the analyte has already been exhaustively extracted in the first extraction. Values above 0.95 indicate that high analyte concentrations are still present in the sample and were not completely extracted during the extraction process.^21,35^

Considering these criteria, the 20 mg mass provided the most suitable conditions for MHS-SPME, enabling adequate decay behavior and appropriate β values while enhancing sensitivity. Thus, 20 mg was selected as the optimal sample mass for subsequent experiments.

### 3.3 Method Validation

Validation must ensure, through experimental studies, that the method meets the requirements of analytical applications, assuring reliable results. The present method was validated for the following parameters: limits of detection (LOD) and quantification (LOQ), linearity, and precision (intraday and interday) according to the SANTE guidelines.^36^

The method demonstrated suitable linearity, with *R* ^2^ values ranging from 0.983 to 0.999, indicating a strong correlation across the tested concentration range. The limit of quantification (LOQ) was defined as the lowest concentration that showed a measurable decay under the MHS conditions. The limits of detection (LOD) and quantification (LOQ) ranged from 0.004 to 0.2 mg·kg^–1^ and from 0.012 to 0.6 mg·kg^–1^, respectively, as shown in Table S3. Both intraday and interday precision were assessed, with relative standard deviations (RSD) consistently below 20%, confirming the method’s reliability and reproducibility (Table S3).

Considering the analytical approaches previously proposed for monitoring these compounds, only two studies have reported the use of MHS for furan quantification in coffee.^37^ In those studies, LOQs ranged from 0.01 to 0.8 mg·kg^–1^, values comparable to those obtained in the present work. Moreover, only one study has applied MHS for the determination of furfural and 5-methylfurfural in wine samples,^38^ reporting LOD of 0.010 mg·kg^–1^ for furfural and 0.002 mg·kg^–1^ for 5-methylfurfural, which are also similar to those reported in the present study.

In contrast, despite the extensive literature on FNOL^39^ in coffee, no studies have reported its quantification by MHS. As furan shows a vapor pressure of 605.2 ± 0.1 mmHg at 25 °C and Furaneol has vapor pressure around 0 mmHg at 25 °C, most methods that proposed the quantification of these compounds use liquid chromatography or derivatization steps previously to chromatographic analysis.^40^ However, coffee is a roasted matrix that enables the application of high temperatures for volatile extraction. Moreover, evidence suggests that MHS can be applied to monitor FNOL, since it was reported in other studies where SPME was used for sample preparation.^41^ Furthermore, the combination of experimental design and a sensitive detection system, such as a high-efficiency source and triple quadrupole detection, enabled the simultaneous quantification of compounds with high and low volatility, such as furan and FNOL. In-house validation further confirmed the method’s accuracy and reliability for quantitative analysis.

### 3.4 Sensory Analysis

Sensory analysis plays a crucial role in the coffee industry by providing valuable feedback to growers, roasters, and producers. It is also essential for quality control and certification to ensure that coffee meets established standards and specifications before reaching consumers.

In the present study, sensory analysis revealed discrepancies between the quality indicated on the packaging and the quality assessed by the trained panel for five samples (S3, S4, S13, S14, and S18) (Table S4). Samples S3 and S4 were marketed as traditional coffee, but they showed an overall quality below 4, with odor and fragrance levels below the recommended standards and numerous defects. As a result, the panel classified them as not recommended for consumption. The same was observed for samples S13, S14, and S18, which were commercialized as superior (S13 and S14) and gourmet (S18) coffees, but the overall quality indicated products with characteristics of traditional and superior coffee, respectively.

Conversely, although it was marketed as gourmet coffee, sample S19 was classified as a specialty coffee by the sensory panel. This classification may result from the combination of high scores for odor, flavor, and body, together with a low level of astringency, defects, and bitterness (Table S3).

Specialty coffee exhibits a more pronounced odor, similar to that of freshly ground coffee. It is also expected to present a balanced profile of acidity, bitterness, and astringency, without sensory defect, characteristics that contribute to higher overall quality.^25,42^ In sample S18, the elevated scores for defects and astringency, combined with lower ratings for odor, flavor, and body, likely contributed to its reduced overall quality. Conversely, samples S3, S4, S13, and S14 showed high scores for astringency and bitterness and low scores for positive attributes such as acidity and flavor, which also resulted in a low overall quality.

The quality of gourmet and specialty coffees results from their production using exclusively 100% Arabica beans of defined origin or blends that meet strict sensory and quality criteria. These coffees must also be free of black, green, and burnt defects, whereas traditional and superior coffees may contain up to 20% and 10% defective beans, respectively.^42^

On the other hand, coffee’s high commercial value makes it particularly susceptible to fraud. Regulatory agencies, industry organizations, and certification agencies rely on quality standards, supply chain traceability, and product testing to detect fraudulent practices. Some adulterants, such as components of the coffee plant itself, are challenging to detect with traditional analytical methods due to the similarity of their chemical composition. To address this, a variety of approaches have been developed, including microscopy-, spectroscopy-, chromatography-, and DNA-based methods.^43^

The addition of impurities or low-cost materials to roasted and ground coffee is one of the most common fraudulent practices. Such adulteration increases product volume but compromises beverage quality, contributing to undesirable flavors and aromas.^44^ Typical adulterantes include corn, barley, chicory and byproducts of the coffee production chain, such as husks.^43^ Barley, corn, chicory, and husks are rich in carbohydrates, which might impact the formation of the furanic compounds during coffee roasting.^45^ This may help explain why one of the samples classified as not recommended for supply showed a high concentration of furanic compounds (sample S3) despite its low sensory quality, as outlined by the sensory panel.

### 3.5 Occurrence

The presence of furans and furanic compounds has been widely reported in various foods, with coffee, which is the second most consumed beverage in the world, showing some of the highest reported levels. Table S5 presents the concentration of the four furan compounds analyzed in commercial samples of ground roasted coffee. The compounds evaluated were detected in all the samples, with levels ranging from 2.89 to 9.90 mg·kg^–1^ for FU, 3.56 to 19.44 mg·kg^–1^ for FURF, 1.72 to 22.86 mg·kg^–1^ for 5-MF, and 0.10 to 2.03 mg·kg^–1^ for FNOL. This high incidence can be explained by the fact that coffee contains several precursors, carbohydrates, and ascorbic acid that can undergo thermal degradation, leading to the formation of furanic compounds.^46^

Some analytes showed concentrations slightly above the maximum levels of the calibration curve, a phenomenon also reported in other studies employing MHS.^21,47,48^ Serrano, Beltrán, and Hernández^48^ demonstrated that extrapolation has no significant impact on the analysis by MHS, especially given that the calculated areas used are theoretical. Therefore, the total area of each compound was determined using the MHS-SPME theory, derived from the analyte area in a single extraction, in conjunction with the calculated β parameter to estimate the concentrations of the compounds in the samples. Therefore, the total for each compound was determined by using MHS-SPME theory, combining the analyte area from a single extraction with the calculated β parameter to estimate sample concentrations.

Higher levels of furanic compounds were detected in gourmet samples, whereas the lowest levels were observed in coffees classified as not recommended for consumption. All quantified compounds showed a positive correlation with the acidity of the coffee beverages (Figure 1). FNOL showed a negative correlation with the bitterness and astringency of the beverages. FNOL has a strong caramel-like odor and can be used to increase the sweetness of food products.^49,50^ Higher levels of FNOL were associated with reduced perception of bitterness and astringency (Figure 1). Similar trend was observed for FURF and 5-MF, which showed significant correlation with all sensory parameters evaluated.

Czerny, Mayer, and Grosch^51^ reported that reducing FNOL levels led to pronounced changes in coffee aroma, emphasizing its critical sensory relevance. Although FURF and 5-MF have been associated with intense bitterness^52^ and identified as precursors in the formation of bitter compounds, such as benzene diols and triols,^53^ they have also been shown to correlate positively with multiple sensory attributes, indicating their broad contribution to coffee quality.^54^

Omission experiments conducted on Yunnan Arabica coffees further confirmed the significant sensory impact of FNOL and 5-MF, reinforcing their essential role in aroma formation and overall coffee quality.^49^

The concentrations of furan obtained in this study were consistent with those reported by Arisseto, Vicente, Ueno, Tfouni, and Toledo^18^ and Zhu, Long, Ma, Huang, Wan, Yu, Xie, and Chen,^55^ who observed FU levels in roasted coffee ranging from 0.911 to 5.85 mg·kg^–1^ and from 0.984 to 6.181 mg·kg^–1^, respectively. Similarly, furfural concentrations were in agreement with those reported by Chaichi, Ghasemzadeh-Mohammadi, Hashemi, and Mohammadi^56^ who found values between 1 and 20 mg·kg^–1^. Slightly higher concentrations of 5-MF were reported in coffees commercialized in China,^57^ and a comparable trend was observed for FNOL in espresso coffee.^58^

A principal component analysis (PCA) was conducted to verify the correlation between the sensory quality of the samples and the levels of FU, FURF, 5-MF, and FNOL. The data was autoscaled, and the classification made for the expert panel was used for labeling the samples as not recommended for consumption, traditional, superior, gourmet, and specialty. With only 2 principal components (PC), it was possible to explain more than 88.2% of the data (Figure 2).

**Figure 2:**
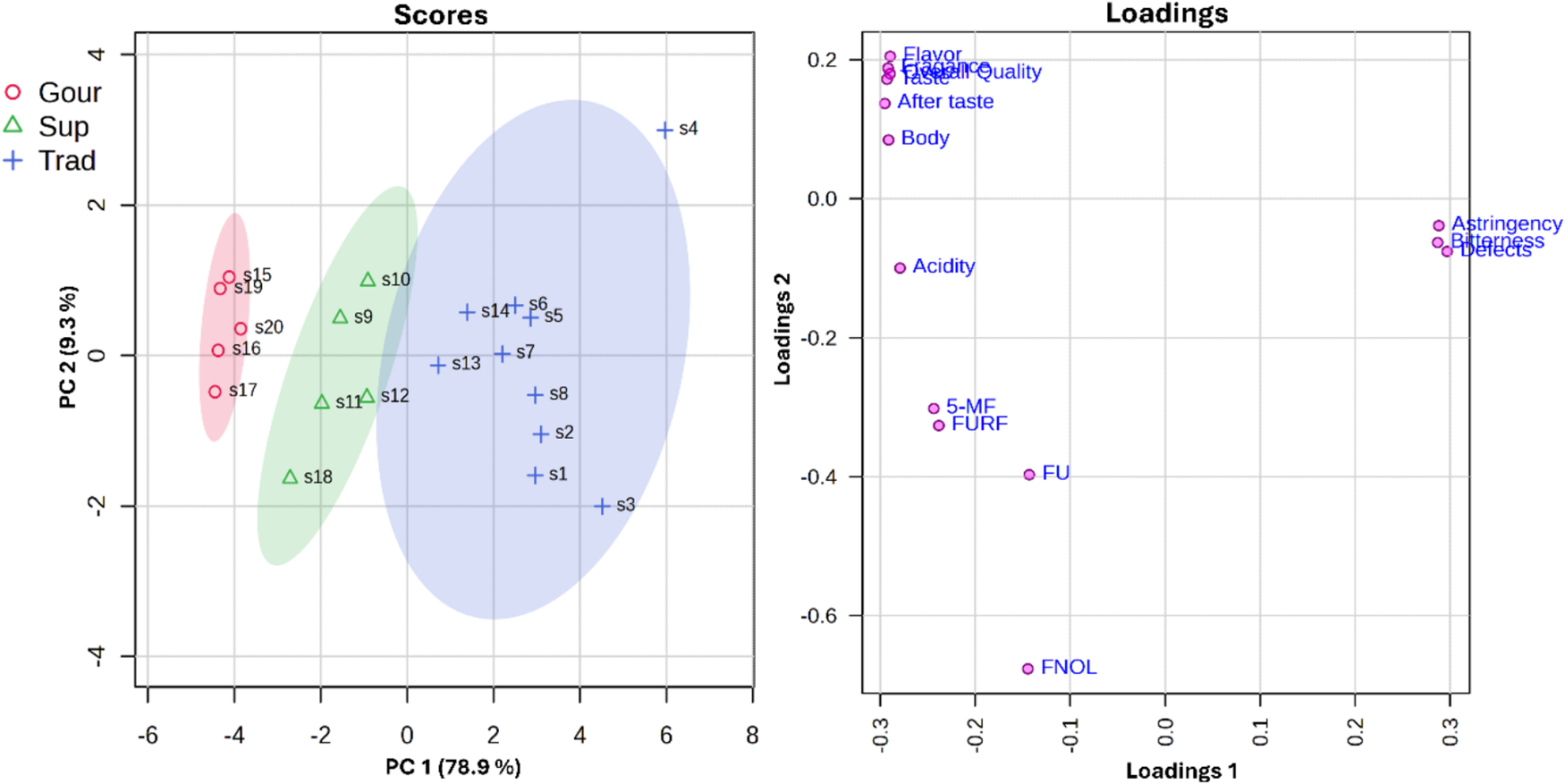
Principal component analysis for sensory parameters and levels of FU, FURF, 5-MF, and FNOL in commercial coffee samples.

The PCA revealed that higher concentrations of these compounds were positively correlated with higher coffee quality. The PCA also highlighted that specialty, gourmet, and traditional coffees have unique characteristics that discriminate them from the other samples. However, for superior coffee, it is possible to detect characteristics between traditional and gourmet samples, which impacts the distribution of these samples between the two groups. This is expected, as superior coffees may contain up to 15% Robusta beans and are blended to meet minimum quality parameters, resulting in a product that achieves a quality between gourmet and traditional coffees.

Specialty and gourmet coffees, on the other hand, are conventionally produced exclusively from Arabica beans and are typically cultivated, harvested, and processed to maximize sugar accumulation to generate a product with a distinct flavor and a high content of sweet flavor notes. During roasting, these sugars contribute to the formation of higher levels of furanic compounds and distinctive flavor profiles.

## 4 Conclusion

For the first time, 5-MF and FNOL were quantified by MHS. Using only 20 mg of sample, the method achieved LOQ varying from 0.012 to 0.6 mg·kg^–1^, good linearity, and satisfactory precision inter and intraday with RDS less than 20%. The proposed method can be seamlessly integrated into routine analytical workflows, offering high throughput and competitive performance for coffee quality assessment.

Sensory analysis revealed that the overall quality of the beverage could be related to the levels of furan compounds in the coffee. Although many studies have investigated strategies to mitigate human exposure to furanic compounds, the present findings highlight their crucial role in shaping coffee aroma and quality. Therefore, future mitigation approaches should aim to reduce furanic levels without compromising the desirable flavor characteristics of the coffee beverage.

## Supplementary Material



## Notes

Sample codes (Table S1), Pareto chart (Figure S1), area decay (Figure S2), and β values (Table S2) for MHS optimization, validation parameters (Table S3), sensory analysis (Table S4), and quantification of furans in commercial coffees (Table S5) (PDF)
