# Supplementary material for: Unveiling the Role of Furanic Compounds in Coffee Quality
Source: ACS Omega. 2026 Apr 8;11(15):23138–47. doi: 10.1021/acsomega.5c13418 (PMC13103814; doi:10.1021/acsomega.5c13418)
Supplement: Supplementary file 1 [file ao5c13418_si_001.pdf]

Supporting information

Unveiling the role of furanic compounds in coffee quality

Valentina Chaves Tognocchi[1], Aline de Oliveira Garcia[1], Fernanda F. G. Dias[2], Silvia Amélia Verdiani Tfouni[1*], Wellington da Silva Oliveira[1,2*]

1. Institute of Food Technology (ITAL), Avenida Brasil 2880, 13070-178, Campinas, SP, Brazil. Centro de Ciência e Qualidade de Alimentos, Instituto de Tecnologia de Alimentos - ITAL, Avenida Brasil n 2880, 13070-178, Campinas, SP, Brazil

2. Department of Food Science and Nutrition, University of Minnesota, St. Paul, MN 55108, United States.

*Corresponding authors:

Wellington da Silva Oliveira

E-mail: wellingtonoliveira1408@gmail.com

Silvia Amélia Verdiani Tfouni

E-mail: tfouni@ital.sp.gov.br

Table S1: Sample codes, commercial classification, and packaging material of the commercial coffee samples analyzed.

Sample	Commercial classification	Package
S1	Extra Strong	Laminated
S2	Extra Strong	Laminated
S3	Traditional	Pouch
S4	Traditional	Pouch
S5	Traditional	Cardboard and laminated
S6	Traditional	Cardboard and laminated
S7	Traditional	Cardboard and laminated
S8	Traditional	Pouch
S9	Superior	Cardboard and laminated
S10	Superior	Cardboard and laminated
S11	Superior	Pouch
S12	Superior	Pouch
S13	Superior	Pouch
S14	Superior	Pouch
S15	Gourmet	Laminated
S16	Gourmet	Laminated
S17	Gourmet	Laminated
S18	Gourmet	Laminated
S19	Gourmet	Laminated with filter
S20	Gourmet	Laminated with filter

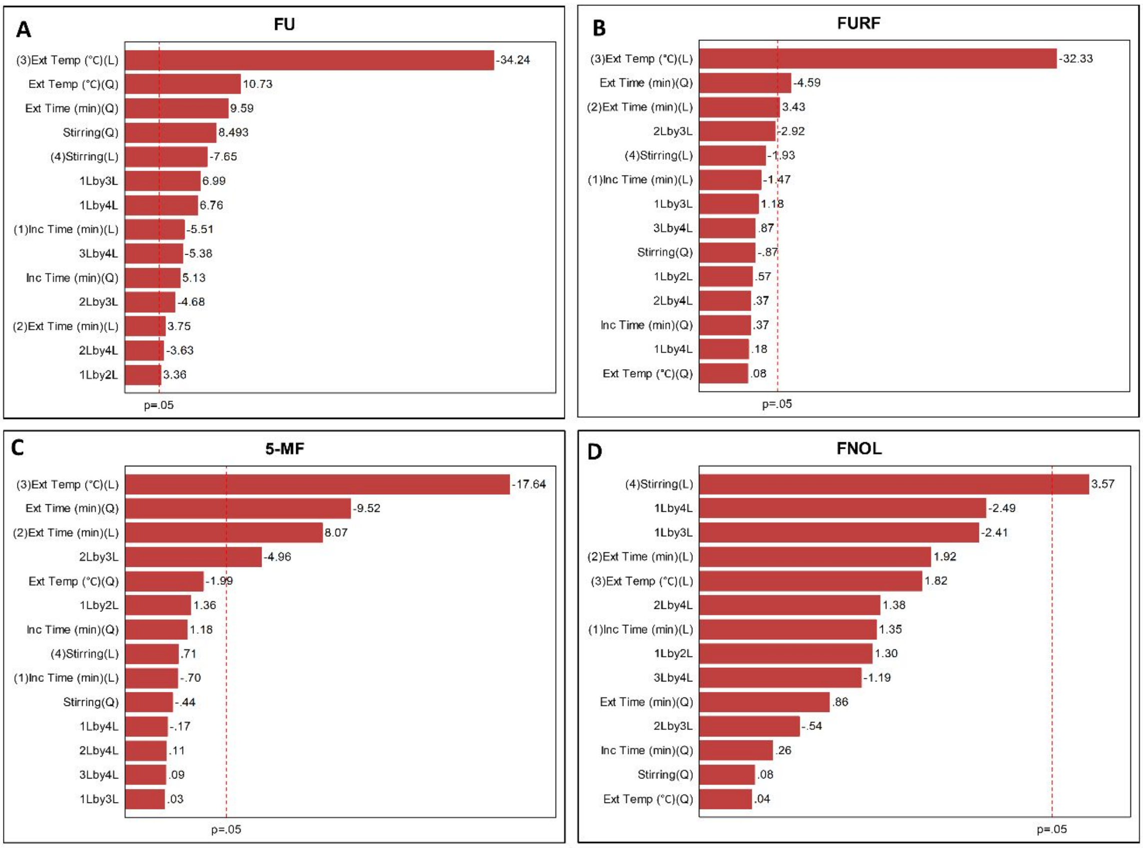

Figure S1. Pareto diagram ($p < 0.05$) obtained from SPME optimization for the extraction of furan FU (A), FURF (B), 5-MF (C), and FNOL (D).

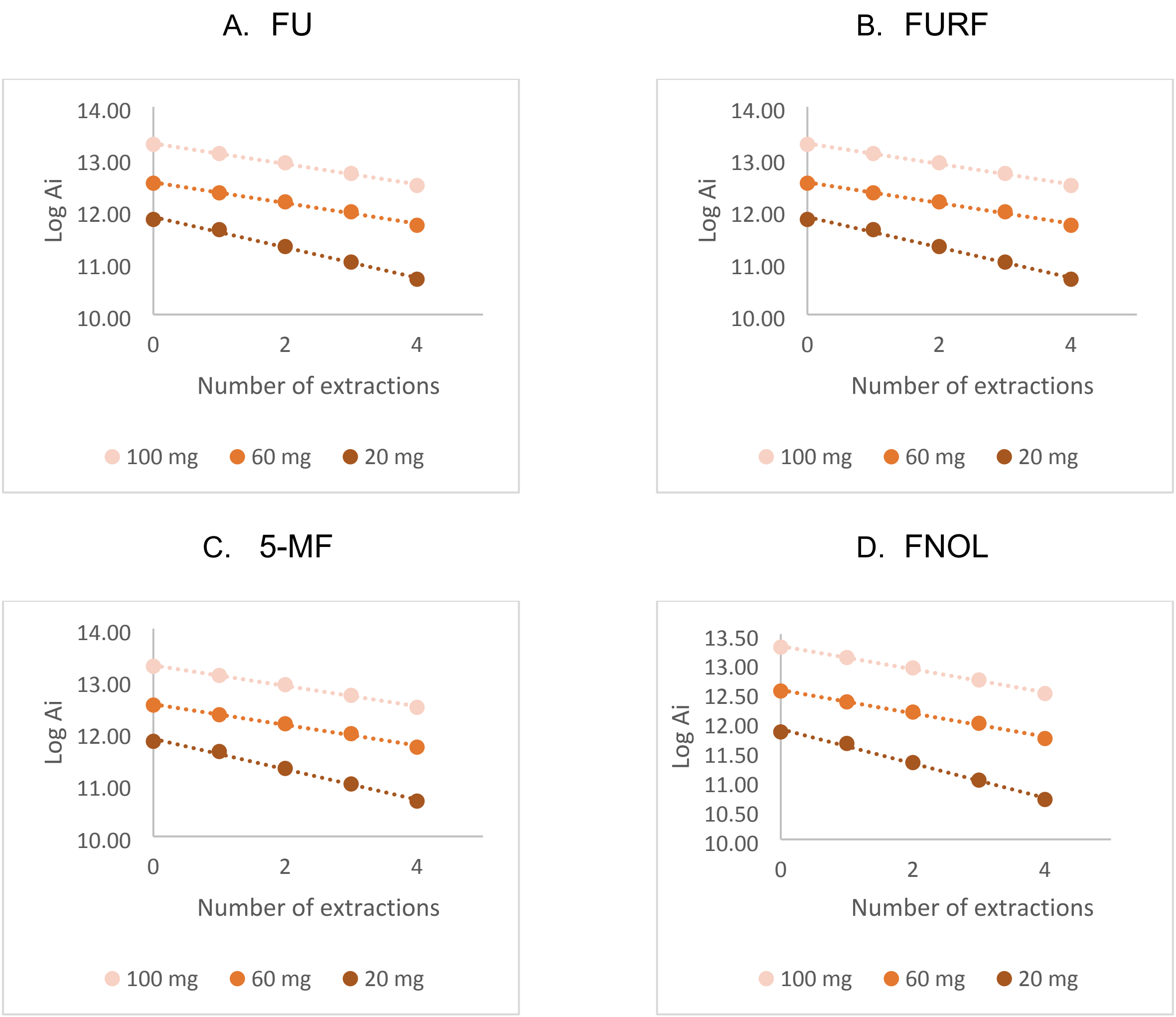

Figure S2. Area decay of the 100, 60 and 20 mg samples. of FU (A), FURF (B), 5-MF (C), and FNOL (D)

Table S2. Comparative data of the use of 20, 60 and 100 mg samples for performing multiple headspace (MHS) extraction.

	Furan	Furfural	5-Methyl furfural	Furaneol
		**20 mg**		
**β**	0.80	0.94	0.79	0.71
**R^2**	0.993	0.982	0.991	0.991
**Curve**	y = -0.2928x + 11.879	y = -0.0586x + 14.889	y = -0.1967x + 14.74	y = -0.2685x + 13.254
		**60 mg**		
**β**	0.84	0.97	0.89	0.84
**R^2**	0.993	0.987	0.982	0.997
**Curve**	y = -0.1985x + 12.545	y = -0.0239x + 15.212	y = -0.0767x + 15.102	y = -0.1336x + 13.501
		**100 mg**		
**β**	0.85	0.98	0.93	0.91
**R^2**	0.996	0.995	0.997	0.996
**Curve**	y = -0.1965x + 13.293	y = -0.0105x + 15.355	y = -0.0436x + 15.181	y = -0.1026x + 13.482

Table S3. Validation parameters ($mg.kg^{-1}$) for the determination of furanic compounds in coffee.

Compounds	LOD	LOQ	Linear Range	Regression Curve	R^2	intraday precision (n=10)			interday precision (n=3)		
						LOQ	Mid-level	Upper-level	LOQ	Mid-level	Upper-level
Furan	0.2	0.6	LOQ-18	$y = 8.7 \times 10^4x + 1.7 \times 10^5$	0.984	3.10	3.44	4.68	3.40	4.57	5.13
Furfural	0.004	0.012	LOQ-22	$y = 3.0 \times 10^6x + 3.0 \times 10^6$	0.983	6.05	3.23	4.54	4.21	4.38	4.98
5-Methyl furfural	0.004	0.012	LOQ-22	$y = 2.9 \times 10^5x + 2.5 \times 10^5$	0.992	8.61	3.32	4.77	7.47	4.71	4.75
Furaneol	0.01	0.03	LOQ-18	$y = 1.1 \times 10^5x - 9.9 \times 10^3$	0.999	18.91	9.47	11.37	17.40	9.59	9.99

Table S4: Attributes and response from the sensory evaluation of commercial coffees.

Sample	Fragrance (powder)	Odor	Defects	Acidity	Bitterness	Flavor	After taste	Astringency	Body	Overall Quality	Sensory Classification
S1	4.5	4.5	5.3	3.2	5.8	4.5	4.5	5.6	5.1	4.5	Traditional
S2	4.7	4.5	5.2	2.8	5.6	4.6	4.6	5.5	4.8	4.6	Traditional
S3	3.4	3.3	6.1	3.1	5.5	3.1	3.7	4.8	5.1	2.8	NR
S4	4.2	4.1	5.8	2.1	5.5	4	3.9	5.6	4.8	3.8	NR
S5	5.06	4.9	4.86	3	5.34	4.92	5	5.36	5.06	4.9	Traditional
S6	5.3	5.2	4.7	3.1	5.2	5	5.1	5.2	5.2	5.1	Traditional
S7	5.06	4.92	4.62	3.1	5.06	5.14	5.24	5.16	5.24	5.24	Traditional
S8	4.62	4.49	5.21	2.87	5.37	4.46	4.59	5.27	5.03	4.41	Traditional
S9	6.42	6.44	2.78	4.36	3.5	6.54	6.5	3.22	6.62	6.42	Superior
S10	6.28	6.32	2.92	4.36	3.8	6.4	6.32	3.26	6.5	6.3	Superior
S11	6.3	6.1	2.3	4.9	3.2	6.5	6.4	2.8	6.3	6.4	Superior
S12	6	5.9	3	4.3	3.8	6	6	3.7	6.2	6	Superior
S13	5.6	5.5	3.8	3.7	5.1	5.5	5.7	4.1	5.7	5.8	Traditional
S14	5.6	5.7	3.6	3	5.6	5.7	5.7	3.8	5.2	5.5	Traditional
S15	7.67	7.58	0.93	4.68	1.95	7.78	7.6	1.07	7	7.77	Gourmet
S16	7.58	7.52	1.06	4.36	2.14	7.74	7.72	1.04	6.94	7.78	Gourmet
S17	7.62	7.56	1.02	4.44	2.2	7.68	7.66	1.1	7.02	7.76	Gourmet
S18	6.4	6.2	1.3	4.4	2.5	6.6	6.5	1.4	6.4	6.2	Superior
S19	7.9	8.1	0.9	4.7	2.5	8.1	8.1	1	7.2	8.1	Specialty
S20	7.5	7.4	1.2	4.3	2.2	7.5	7.5	1.1	6.9	7.5	Gourmet

NR: not recommended for consumption

Table S5. Occurrence of furan compounds in commercial coffee samples.

Sample	Furan	Furfural	5-methyfurfural	Furaneol
S1	6.67 ± 0.28 C-F	11.64 ± 0.30 GHI	14.04 ± 0.42 FG	1.65 ± 0.10 ABC
S2	7.12 ± 0.60 CD	11.89 ± 0.28 GH	13.78 ± 0.27 GH	1.26 ± 0.19 D-E
S3*	6.17 ± 0.71 D-H	10.71 ± 0.43 IJ	10.24 ± 0.32 I	1.69 ± 0.51 AB
S4*	2.89 ± 0.21 I	3.56 ± 0.04 K	1.72 ± 0.06 J	0.10 ± 0.001 F
S5	5.13 ± 0.24 GH	10.32 ± 0.20 J	13.99 ± 0.28 GH	0.85 ± 0.02 E
S6	5.62 ± 0.12 D-H	10.84 ± 0.27 HIJ	12.81 ± 0.30 H	0.76 ± 0.03 E
S7	6.00 ± 0.61 D-H	11.49 ± 0.09 GHI	15.19 ± 0.18 EF	0.98 ± 0.07 DE
S8	5.31 ± 0.84 FGH	13.74 ± 0.78 DE	17.74 ± 0.89 D	0.96 ± 0.53 DE
S9	6.74 ± 0.17 C-F	16.10 ± 0.43 B	19.29 ± 0.45 C	0.78 ± 0.11 E
S10	6.34 ± 0.81 C-H	11.98 ± 0.30 FG	18.74 ± 0.18 CD	0.72 ± 0.05 EF
S11	7.84 ± 0.73 BC	14.64 ± 0.18 CD	21.98 ± 0.29 A	1.23 ± 0.05 D-E
S12	5.90 ± 0.18 D-H	16.36 ± 0.22 B	22.41 ± 0.20 A	1.25 ± 0.08 D-E
S13	6.63 ± 0.55 C-G	13.02 ± 0.21 EF	17.99 ± 0.14 D	1.00 ± 0.03 CDE
S14	5.82 ± 0.48 D-H	12.07 ± 0.60 FG	16.37 ± 0.68 E	0.83 ± 0.25 E
S15	5.05 ± 0.20 H	16.60 ± 0.28 B	20.64 ± 0.25 B	1.29 ± 0.04 D-E
S16	5.79 ± 0.41 D-H	19.44 ± 0.22 A	22.86 ± 0.32 A	1.55 ± 0.23 A-D
S17	5.55 ± 0.24 E-H	18.70 ± 0.29 A	22.15 ± 0.34 A	2.03 ± 0.04 A
S18	8.77 ± 0.68 AB	14.83 ± 0.35 C	21.94 ± 0.41 A	1.85 ± 0.10 AB
S19	9.90 ± 0.47 A	11.38 ± 0.38 G-J	14.65 ± 0.46 FG	1.22 ± 0.37 D-E
S20	6.92 ± 0.14 CDE	15.64 ± 0.32 BC	22.20 ± 0.28 A	1.33 ± 0.01 D-E

* Classified as coffee not recommended for consumption; Means (n = 3) in each column with different letters are significantly different, according to the Tukey test ($p < 0.05$).
